# Low-Molecular-Weightt Polysaccharides From *Pyropia yezoensis* Enhance Tolerance of Wheat Seedlings (*Triticum aestivum L.)* to Salt Stress

**DOI:** 10.3389/fpls.2018.00427

**Published:** 2018-04-17

**Authors:** Ping Zou, Xueli Lu, Changliang Jing, Yuan Yuan, Yi Lu, Chengsheng Zhang, Lei Meng, Hongtao Zhao, Yiqiang Li

**Affiliations:** ^1^Marine Agriculture Research Center, Tobacco Research Institute of Chinese Academy of Agricultural Sciences, Qingdao, China; ^2^College of Agriculture, Qingdao Agricultural University, Qingdao, China; ^3^State Key Laboratory of Bioactive Seaweed Substances, Qingdao, China

**Keywords:** *Pyropia yezoensis*, polysaccharides, molecular weight, salt stress, antioxidant enzyme activities

## Abstract

Soil salinity is one of the major issues worldwide that affects plant growth and reduces agricultural productivity. Seaweed polysaccharides have been shown to promote crop growth and improve the resistance of plant to abiotic stresses. *Pyropia yezoensis* is a commercially important edible red alga in Southeast Asia. However, there is little research on the application of polysaccharides from *P. yezoensis* in agriculture. The molecular weight (MW) of polysaccharides influences their properties. Therefore, in this study, four representative polysaccharides from *P. yezoensis* (PP) with different MWs (MW: 3.2, 10.5, 29.0, and 48.8 kDa) were prepared by microwave-assisted acid hydrolysis. The relationship between the degradation of polysaccharides from *P. yezoensis* (DPP) and their effects on plant salt tolerance was investigated. The results showed that exogenous PP and DPPs increased wheat seedling shoot and root lengths, and fresh and dry weights, alleviated membrane lipid peroxidation, increased the chlorophyll content and enhanced antioxidant activities. The expression level examination analysis of several Na^+^/K^+^ transporter genes suggested that DPPs could protect plants from the damage of salt stress by coordinating the efflux and compartmentation of Na^+^. The results demonstrated that polysaccharides could regulate antioxidant enzyme activities and modulate intracellular ion concentration, thereby to protect plants from salt stress damage. Furthermore, there was a significant correlation between the tolerance of wheat seedlings to salt stress and MW of polysaccharides. The results suggested that the lower-MW samples (DPP1, 3.2 kDa) most effectively protect wheat seedlings against salt stress.

## Introduction

Soil salinization is a major concern in agriculture. It is estimated that by 2050, salinization will degrade up to 30% of all cultivated land (Van Oosten et al., 2017). High salinity is a major abiotic stress factor that restricts crop growth and yield worldwide. Salinity reduces photosynthesis, plant growth and development in plants by causing osmotic stress, ion injury or oxidative stress (Rivero et al., 2014). Plants have developed several comprehensive mechanisms to tolerate or overcome the effects of salinity (Bose et al., 2014). The application of exogenous biostimulators may effectively protect plants against salinity.

*Pyropia yezoensis* is a commercially important edible red alga in Southeast Asia. It is cultivated and consumed in China, Japan, and South Korea. The chemical structure of *P. yezoensis* polysaccharides was investigated by Zhou et al. (2012). It has a backbone of alternating (1 → 4) -3,6-anhydro-α-L-galactopyranose units and (1 → 3) - linked β-D-galactose or (1 → 4)-linked α-L-galactose 6-sulfate units. In recent years, *P. yezoensis* polysaccharides have attracted attention as biomedical material because of their and antioxidants (Zhao et al., 2006; Zhou et al., 2012), anticancer (Yu et al., 2015), hypolipidemic (Qian et al., 2014), immunoregulatory (Zhang et al., 2003), the neurotrophic and neuroprotective activities (Mohibbullah et al., 2015). Moreover, it has been demonstrated that polysaccharides carrageenans and their oligosaccharides can trigger defense systems against plant pathogens including bacteria, virus and fungi (Stadnik and de Freitas, 2014). Sangha et al. (2010) found that λ-carrageenan can activate defense mechanisms of *Arabidopsis thaliana* (L.) Heynhold against *Sclerotinia sclerotiorum*(Libert) de Baryvia an increase in oxalate oxidase activity and the genes expression associated with jasmonic acid signaling. Vera et al. (2012) demonstrated the application of κ-, ι-, and λ-oligo-carrageenans resulted in a durable protection against tobacco mosaic virus (TMV), *Botrytis cinerea* Persoon and *Pectobacterium carotovorum* (Jones) Waldee.

The activities of polysaccharides depend on the molecular weight (MW), the number of sulfate groups and the molar ratio of sulfate/total sugar. The MW of polysaccharides influences their physicochemical behavior. It may be difficult for high-MW crude polysaccharides to permeate the basement membrane and exert its effects *in vivo*. In contrast, a low-MW polysaccharide prepared from *Laminaria japonica* has effective scavenging activities on superoxide radical, hydroxyl radical, and hypochlorous acid *in vitro* (Zha et al., 2016). Zhou’s work indicated that the ultrasonic degraded polysaccharide from *Porphyra yezoensis* exhibited stronger antioxidant activity than that of the natural polysaccharide (Zhou et al., 2012). These results indicate that polysaccharide antioxidant activity may be inversely proportional to MW. The aim of this paper was to study the relationship between MW of polysaccharides from *P. yezoensis* and their activities of improving plant’s tolerance to salt stress. Therefore, the polysaccharides were partially degraded in the aqueous phase with the assistance of microwaves. Then the effects of four degraded and natural polysaccharides on wheat seedlings under salt stress were investigated. These polysaccharides may represent a potentially simple, efficacious, and sustainable approach for inhibiting the harm to commercially important crops caused by abiotic stress.

## Materials and Methods

### Materials and Equipments

In February 2017, *P. yezoensis* individuals were collected from a farming raft in Rizhao, Shandong Province, China. These *P. yezoensis* individuals were washed twice with fresh water, flash-frozen, and stored at -20°C. The frozen samples were lyophilised and ground in a mill. The dried sample powder was stored at 4°C.

Standard sugars, Nitro blue terazolium (NBT), hydrogen peroxide (H_2_O_2_), trichloroacetic acid (TCA) were purchased from Sigma Chemicals Co. All other reagents were of analytical grade. Microwave synthesis/extraction reaction station (Type: MAS-II) was purchased from Shanghai SINEO Microwave Chemistry Technology Co., Ltd. (China).

### Preparation of Polysaccharide Fraction From *P. yezoensis*

Dry algae powder (100 g) was extracted with distilled water (4 L) at 100°C for 4 h. The slurry was filtered through gauze and the liquid supernatant was filtered using siliceous earth. The filtrate was dialyzed against distilled water for 48 h then the solution was concentrated to approximately ¼ of its original volume under reduced pressure. The polysaccharide was precipitated using four times volume of ethanol and then lyophilized to yield white powdered products and referred as PP.

To prepare the polysaccharide fraction, a reaction solution (2% w/v) was prepared by dissolving PP (2.0 g) in distilled water (100 mL). Different volumes of hydrogen chloride (HCl) were added to the solution at various temperatures and time intervals (Li et al., 2013). The mixtures were exposed to microwave irradiation with constant magnetic stirring and temperature monitoring with an infrared thermometer in a laboratory microwave reaction station. The reaction mixture was then removed and subjected to gel permeation chromatography. Four degraded polysaccharides, namely, DPP1, DPP2, DPP3, and DPP4, were prepared according to the conditions listed in **Supplementary Table S1**. After the reaction was complete, the solution was precipitated with ethanol then washed with it twice.

### Chemical Analysis of Four Degraded and Natural Polysaccharides

Polysaccharide MW were determined using an Agilent 1260 gel permeation chromatograph (Agilent Technologies, United States) fitted with a refractive index detector. The chromatography was run on a TSK G4000-PWxl column with 0.05 M aqueous NaNO_3_ as the mobile phase. The flow rate was 0.5 mL/min and the column temperature was 30°C. The standards used to calibrate the column were dextrans MW 1000, 5000, 12,000, 25,000, 50,000, 80,000, 270,000, and 670,000 Da (Sigma, United States).

The total sugar content was determined by the phenol-sulphuric acid method using D-galactose as the reference standard. The sulfate content was determined by the barium chloride (BaCl_2_) method (Zhao et al., 2006). The protein content was measured by the Bradford method using bovine serum albumin (BSA) as the reference standard (Mishra et al., 2013). Uronic acid was estimated from a modified carbazole method using glucuronic acid as the reference standard (Hou et al., 2012). Fourier transform infrared (FT-IR) spectra of the polysaccharides were plotted with a Thermo Fisher Scientific Nicolet iS10 FT-IR spectrometer in potassium bromide (KBr) disks.

### Effect of Polysaccharides on Wheat Seedlings Under Salt Stress

#### Plant Material and Treatments

Wheat (*Triticum aestivum* L. Jimai 22) seeds were surface-sterilized with a 1% (v/v) sodium hypochlorite solution for 10 min and thoroughly rinsed with distilled water. Seeds were germinated for 24 h at 25°C and then sown on Petri dishes. Germinated seeds were sown on Petri dishes containing nylon mesh and Hoagland solution. The plates were placed in a growth incubator under the following controlled environmental conditions: a 14 h/10 h photoperiod, a 25°C daytime/20°C night time cycle, a 65% relative humidity, and a photosynthetic photon flux intensity of 800 μmol m^-2^ s^-1^. When the second leaves were fully expanded, the wheat seedlings were randomly divided into seven groups. Seven treatments included a control check (neither PP nor NaCl), 100 mM NaCl alone as a negative control, 5 different PP-NaCl mixtures [0.01% (w/v) DPP of 3.2 kDa, 10.5 kDa, 29.0 kDa, 48.8 kDa, and PP of 370.5 kDa). There were 40 plants in every petri dishes and each group contained three petri dishes. The nutrient solutions were renewed every 1 day.

#### Growth Parameters

After 10 days of salt stress, three samples were randomly selected from each group and their physiological indexes were determined. After that, 30 plants were randomly chosen and harvested to measure their shoot lengths, root lengths, and fresh weight. Samples were then dried at 105°C for 2 h to determine the dry weights.

#### Determination of Lipid Peroxidation and Membrane Permeability

Membrane permeability was assessed by measuring the relative electric leakage (REL) of the second fully expanded leaf (Li et al., 2017). Leaves (1.0 g) were cut into 0.5-cm pieces and placed in a 50 mL test tube containing 30 mL distilled water. Afterward, the leaf samples in test tubes were vacuumed for 30 min, immersed and vibrated for 20 min, and then measured the conductivity of the solution (EC1) (DDSJ-308A, Shanghai Instrument and Electrical Scientific Instrument Ltd. Shanghai, China). Samples were then boiled for 30 min. When the solution was cooled to room temperature, the conductivity (EC2) was measured again. REL was calculated as EC1/EC2 × 100%.

The malondialdehyde (MDA) content in plants indicates the level of lipid peroxidation. MDA levels were determined using a thiobarbituric acid (TBA) reaction (Zhang et al., 2016). After 10 days NaCl treatment, 0.5 g leaf samples were homogenized in 10% (w/v) trichloroacetic acid (TCA). The homogenates were then centrifuged at 4,000 × *g* for 10 min. Two milliliters of 0.6% (w/v) TBA was added to 2 mL of the supernatant. The mixture was heated in boiling water for 15 min and cooled immediately afterward. The mixture was centrifuged at 10,000 × *g* for 15 min. Absorbances (optical densities) were read at 450, 532, and 600 nm. The MDA content was recorded as μg MDA/g FW.

#### Chlorophyll Contents and Photosynthetic Characters

After a 10-days NaCl treatment, the chlorophyll a (Chl a), chlorophyll b (Chl b) and total chlorophyll (Chla+b) content in the seedlings were measured in 95% ethanol (Ma et al., 2017). The chlorophyll content was measured in a spectrophotometer at 665 and 649 nm. The entire procedure was performed under subdued light to avoid chlorophyll degradation. Transpiration rate (E), photosynthetic rate (Pn), stomatal conductance (gs) and intercellular CO_2_ concentration (Ci) were determined with a portable photosynthesis system (L.MAN-LCPro-SD, BioScientific Ltd., United Kingdom). Atmospheric conditions were as follows: 25 ± 2°C, gas flow rate of 200 μmol s^-1^, photosynthetic photon flux density of 800 μmol m^-2^s^-1^ and CO_2_ concentration of 400 ± 5 μmol m^-2^s^-1^.

#### Soluble Sugar Content and Proline Content

The soluble sugar was measured as follows: 0.5-g leaf samples were chopped and heated at 100°C in 5 mL distilled water for 30 min. The extract was diluted 5×. Extract aliquots of 500 μL, 1 mL of 5% (v/v) phenol, and 5 mL of sulphuric acid were mixed and left to stand 5 min. The absorbance was then read at 485 nm. The soluble sugar concentration was quantified by comparison against a glucose standard curve.

The proline content was determined by grinding 0.2-g leaf samples in liquid nitrogen and homogenizing them in 5 mL of 3% (w/v) sulphosalicylic acid (Huang et al., 2015). After heating at 100°C for 10 min, the homogenate was cooled to room temperature and centrifuged at 5,000 × *g* for 4 min. The proline content in the supernatant was determined spectrophotometrically at 520 nm.

#### Antioxidant Enzyme Activities

After a 10-days salt stress, 0.5-g samples of the second fully expanded leaves were used to extract enzymes. The samples were homogenized in liquid nitrogen and brought up to a volume of 5 mL with cold sodium phosphate buffer solution (pH 7.8). The homogenates were centrifuged at 12,000 × *g* and 4°C for 15 min. The supernatants were immediately used in the determination of enzyme activities.

The total soluble protein was determined by the Bradford method (Huang et al., 2015). A 100-μL aliquot of supernatant and 5 mL of Coomassie Brilliant Blue G-250 were mixed, and the absorbance was read at 595 nm. The protein concentration was quantified by comparison with a standard curve using BSA.

The superoxide dismutase (SOD) activity was assayed by the extent to which it inhibited the photochemical reduction of β-nitro blue tetrazolium chloride (NBT) (Qiu et al., 2014). The 3 mL reaction mixture consisted of 0.1 mL enzyme extract, 50 mM phosphate buffer (pH7.8), 0.1 m MEDTA,130 mM methionine,0.75 mM NBT, and 0.02 mM riboflavin. Their action was initiated by placing the tubes under two 40W fluorescent lamps. After 10 min, the reaction tubes were removed from the light source. Non-illuminated and illuminated reactions without supernatant served as calibration standards. One unit of SOD was defined as the amount of enzyme needed to inhibit NBT reduction by 50% at 560 nm.

The activity of CAT was determined from the rate of disappearance of H_2_O_2_ as measured by the decline in the absorbance at 240 nm (Zou et al., 2016). CAT activity was assayed in a 3-mL reaction mixture containing 0.1 mL of enzyme extract, 50 mM phosphate buffer (pH7.8) and 0.2 percent H_2_O_2_. The decomposition of H_2_O_2_ was measured by following the decline in absorbance at 240 nm for 3 min and the catalase (CAT) activity was expressed as H_2_O_2_ reduced min^-1^mg^-1^ protein.

The peroxidase (POD) activity was determined via the method described by Rasool et al. (2012). The reaction mixture contained 0.05 mL enzyme extract, 0.95 mL guaiacol solution and 2 mL 0.2% H_2_O_2_ solution in phosphate buffer solution. The increase in absorbance was recorded at 470 nm for 3 min. The POD activity was calculated from the rate of the formation of the guaiacol dehydrogenation product and was expressed as μmol GDHP min^-1^ mg^-1^ protein.

#### Measurement of Na^+^ and K^+^ Concentration

The measurement of Na^+^ and K^+^ concentration has been described previously (Zhang et al., 2018). The plant tissues, leaves, sheaths, and roots were dried at 60°C overnight. The dry samples (0.5 g) were incinerated in a muffle furnace at 500°C for 6 h. The ashes were dissolved in 5 ml concentrated nitric acid and volumed with distilled water at 500 ml. The Na^+^ and K^+^ concentrations were determined using an Atomic Absorption Spectrometer 900T (PerkinElmer, United States).

#### Expression Analysis of Genes Encoding Na^+^/K^+^ Transporter

Total RNA was extracted from the leaves, sheaths, and roots of the wheat seedlings using Plant RNA Extraction Kit (Takara, Dalian, China). Total RNA was quantified by UV spectrophotometer. First-strand cDNA was synthesized by PrimeScript^TM^ RT Reagent Kit with gDNA Eraser (Takara, Dalian, China). qRT-PCR was performed in an ABI 7500 [Life Tech (Applied Biosystems), United States] using the TB Green^TM^ *Premix Ex Taq^TM^* (Takara, Dalian, China). The PCR products were amplified and detected via a melting curve analysis. The expression level of gene was analyzed using comparative threshold cycle method (2^-ΔΔ^*^C^*^t^) with β-actin as the control. The sequences of the primers used were listed in **Supplementary Table S2**.

### Statistical Analysis

Each test was performed randomly in triplicate, each of the data points was expressed as the average ± SD of three independent replicates. Data were subjected to ANOVA analysis by SPSS (version 19.0) and Duncan’s test (*P* < 0.05) to compare the mean value of different treatments.

## Results and Discussion

### Chemical Analysis of the Four Degraded and Natural Polysaccharides From *P. yezoensis*

The results of the chemical analyses are shown in **Table 1** and **Supplementary Figure S1**. Four degraded products had sulfate and protein contents similar to that of natural PP. The results indicated that free radical degradation could not cause desulphation. Though the total sugar and uronic acid contents of degraded products were lower than that of natural PP, there were no significant differences among the four degraded polysaccharides.

**Table 1:** Properties of four degraded (DPP1,2,3,4) and natural polysaccharides (PP) from *P. yezoensis*.

Sample	MW (kDa)	Total sugar (%)	Uronic acid (%)	Protein (%)	Sulfate (%)
PP	370.5	77.7 ± 1.5^a^	9.1 ± 0.1^a^	3.6 ± 0.1	19.8 ± 1.4
DPP1	3.2	57.4 ± 1.8^b^	4.1 ± 0.1^b^	3.3 ± 0.1	21.2 ± 2.3
DPP2	10.5	58.0 ± 6.7^b^	4.5 ± 0.2^b^	3.3 ± 0.1	20.9 ± 2.4
DPP3	29.0	56.4 ± 2.9^b^	4.8 ± 0.3^b^	3.4 ± 0.3	21.3 ± 2.1
DPP4	48.8	57.6 ± 3.2^b^	4.7 ± 0.6^b^	3.5 ± 0.1	21.9 ± 2.3

*Values are mean ± SD of three replicates. Different letters indicate significant differences at P < 0.05*.

**FIGURE 1:**
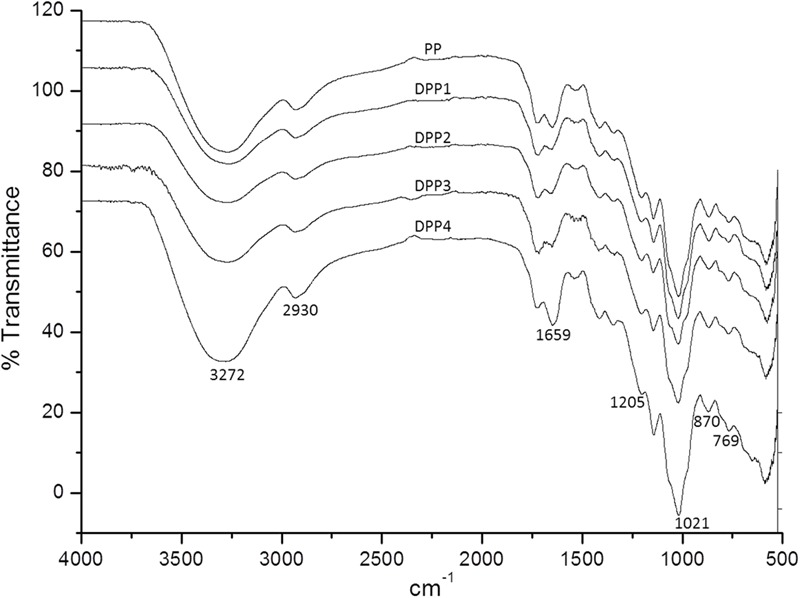
FT-IR spectra of four degraded (DPP1,2,3,4) and natural polysaccharides (PP) from *P. yezoensis*.

The FT-IR spectra for natural and degraded *P. yezoensis* polysaccharides are shown in **Figure 1**. Typical polysaccharides absorptions at 3272, 2930, 1659, 1205, 1021, 870, and 769 cm^-1^ were evident in all the samples. Absorption at 1,205 cm^-1^ represented a sulfate moiety from the stretching of the S = O bond. The signal at 769 cm^-1^ indicated a sulfate attached to a primary hydroxyl group (Zhao et al., 2006). The weak peak at 870 cm^-1^ was due to the 3,6-anhydro-galactose unites in the polysaccharide. The peak at 1659 cm^-1^ appeared was assigned to the carboxylic group. These results suggested that no major functional group transformations occurred in the degradation process.

Microwave irradiation has been recently considered for the degradation of polymer compounds because it reduces the MW simply by breaking the weakest chemical bonds without altering the chemical nature of the polymer (Hou et al., 2012; Li et al., 2013).

### Effect of Polysaccharides on Wheat Seedlings Under Salt Stress

#### Plant Growth and Biomass Accumulation

Increased soil salinity can cause abiotic stress and significantly inhibit crop germination, growth, and yield. As shown in **Table 2**, under NaCl stress, the root- and shoot lengths and the fresh- and dry weights of wheat seedlings were all significantly depressed (*P* < 0.05) relative to the untreated control. In contrast, all of these parameters increased relative to the negative control in plants treated with PPs of different MW.

**Table 2:** Effects of four degraded (DPP1,2,3,4) and natural polysaccharides (PP) from *P. yezoensis* on growth parameters of wheat seedlings.

	Shoot length (cm)	Root length (cm)	Wet weight (g)	Dry weight (g)
Control	21.6 ± 1.8^a^	23.5 ± 3.2^a^	0.622 ± 0.189^b^	0.086 ± 0.017^b^
NaCl stress	20.2 ± 2.2^b^	21.2 ± 2.0^b^	0.538 ± 0.147^c^	0.068 ± 0.015^d^
DPP1 + NaCl stress	21.2 ± 1.0^a^	23.2 ± 2.4^a^	0.756 ± 0.113^a^	0.104 ± 0.015^a^
DPP2 + NaCl stress	21.3 ± 1.5^a^	22.2 ± 2.0^ab^	0.647 ± 0.100^b^	0.086 ± 0.014^b^
DPP3 + NaCl stress	21.1 ± 1.7^a^	22.3 ± 1.8^ab^	0.640 ± 0.095^b^	0.086 ± 0.013^b^
DPP4 + NaCl stress	21.2 ± 1.7^a^	22.5 ± 1.6^ab^	0.642 ± 0.103^b^	0.088 ± 0.012^b^
PP + NaCl stress	21.4 ± 1.8^a^	22.4 ± 2.6^ab^	0.643 ± 0.104^b^	0.077 ± 0.014^c^

*Values are mean ± SD of 30 replicates. Different letters indicate significant differences at P < 0.05.*

Compared with the NaCl stress treatment, the exogenous application of DPPs and PP increased wheat seedling shoot lengths by 4.9, 5.2, 4.5, 5.0, and 6.1%, respectively (*P* < 0.05). Nevertheless, there were no statistically significant differences in wheat seedling shoot lengths among the PP treatments. Similarly, relative to the negative control, the application of DPPs and PP increased wheat seedling root lengths by 9.5, 5.0, 5.1, 6.1, and 5.6%, respectively (*P* < 0.05). PPs also increased the fresh- and dry weights of wheat seedlings under NaCl stress. DPPs and PP increased the wheat seedling fresh weights by 40.6, 20.2, 18.9, 19.4, and 19.5% and their dry weights by 52.5, 25.9, 26.1, 29.1, and 13.7%, respectively, relative to the negative control. The fresh- and dry seedling weights in the DPP1 group were significantly higher than those of the other groups. On the basis of these results, it could be suggested that the application of any of the PPs could improve the wheat seedling growth parameters tested in this study. Of the five PPs applied, DPP1 (3.2 kDa) was the most effective at improving the growth parameters of plants under salt stress.

#### Lipid Peroxidation

**FIGURE 2:**
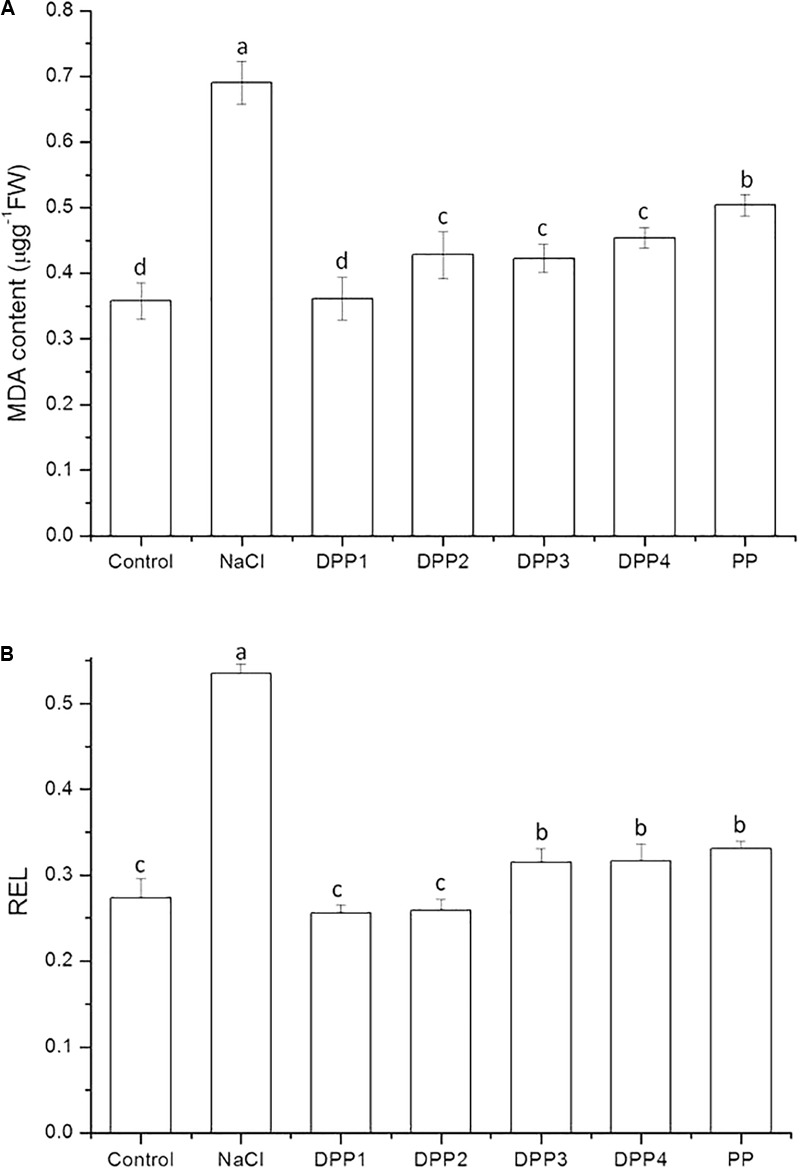
Effect of four degraded (DPP1,2,3,4) and natural polysaccharides (PP) from *P. yezoensis* on MDA content **(A)** and REL **(B)** in leaves of Jimai-22. Values are the mean ± SD of three replicates. Different letters indicate significant differences at *P* < 0.05.

As a product of lipid peroxidation, MDA is an indicator of oxidative free radical damage to cell membranes. The results showed that the MDA content in the leaves of the wheat seedlings (**Figure 2A**) increased significantly by 93.0% in response to NaCl stress (*P* < 0.05). PPs of different MW reduced the MDA content to 47.6, 38.0, 38.7, 34.3, and 27.0% (*P* < 0.05) compared to NaCl-stressed plants. Similarly, the relative electric leakage (REL) significantly increased by 95.3% due to NaCl stress (*P* < 0.05, **Figure 2B**). PPs of different MW reduced the REL to 52.2, 51.6, 41.2, 40.8, and 38.1% (*P* < 0.05) relative to NaCl-stressed plants. Exogenous PPs treatment may mitigate NaCl-induced oxidative damage. PPs could scavenge free radicals and prevent lipid peroxidation caused by the active oxygen produced under salt stress (Bose et al., 2014). Moreover, DPP1 reduced the MDA content in the seedlings relative to NaCl-stressed plants significantly more than the other DPPs and PP (*P* < 0.05). Nevertheless, there were no statistically significant differences in terms of MDA content reduction among DPP2, DPP3, and DPP4.

#### Chlorophyll Content and Photosynthetic Characters

**FIGURE 3:**
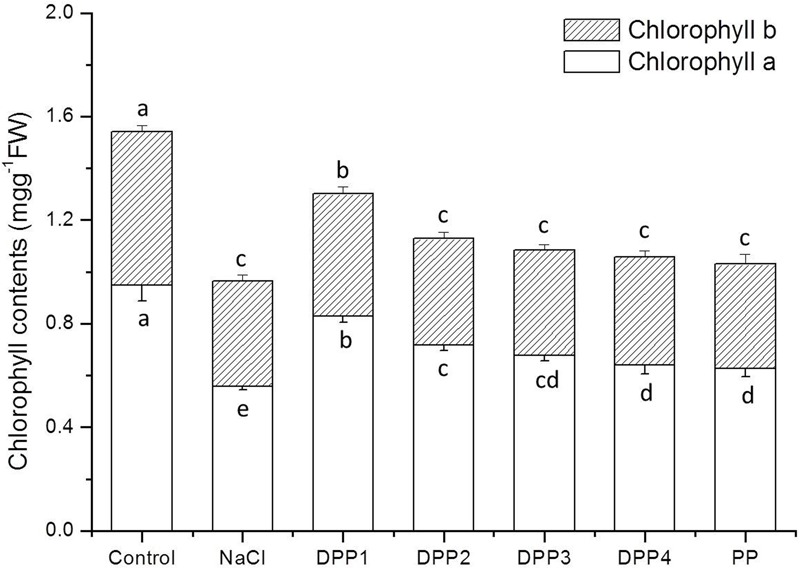
Effect of four degraded (DPP1,2,3,4) and natural polysaccharides (PP) from *P. yezoensis* on chlorophyll content in wheat seedlings. Values are the mean ± SD of three replicates. Different letters indicate significant differences at *P* < 0.05.

Chlorophyll content is widely used as an important indicator of abiotic stress tolerance in plants. The chlorophyll concentration in plants exposed to stressors like salinity tends to be significantly lower than that of control plants (Sudhir and Murthy, 2004). In this study, the Chl-a and Chl-b contents significantly decreased under NaCl stress by 41.1% (*P* < 0.05) and 31.6% (*P* < 0.05), respectively, in comparison with the control (**Figure 3**). The Chl-a contents of plants treated with PPs of different MW were 48.3, 28.7, 21.4, 14.7, and 12.1% greater than that of the negative control (*P* < 0.05). In contrast, DPP1 significantly increased Chl-b content by 16.5% relative to the salt-stressed plants whereas the Chl-b contents in the plants treated with DPP2, DPP3, DPP4, and PP did not significantly differ from those of the NaCl-stressed group.

Improvement in the growth of plants which are subjected to various stressors and are receiving PPs treatment is determined by the increases in chlorophyll content and photosynthetic efficiency (Mehta et al., 2010). In the present study, the salt-stressed plants exhibited significant decreases in Chl-a, Chl-b, and total chlorophyll contents compared with the control. On the other hand, the PPs with different MW significantly increased chlorophyll levels in plants under NaCl stress. The results suggested that PPs protected chlorophyll from degradation in salt-stressed wheat seedling leaves.

It is generally recognized that declined photosynthesis by salt stress is mainly due to stomatal closure, feedback inhibition because of reduced sink activity, and swelling or direct effect of salt stress on stomatal conductance (Zahra et al., 2014). The results showed that gs significantly reduced under salt stress (*P* < 0.05) (**Table 3**), which was caused by an excessive accumulation of Na^+^ ion in the guard cells. And it leaded to a decrease in available Ci as the results showed. Closure of stomata and reduction of the availability of Ci resulted in a Pn reduction. In this work, salt-stressed wheat seedlings had a 30.8% reduction in Pn compared with control (*P* < 0.05), which is in accord with previous findings (Zou et al., 2015). When treated with DPPs and PP with different MWs, the value of Pn increased by 128.6, 93.9, 92.7, 68.4, and 54.9%, separately (*P* < 0.05). The responding trends of gs and E were similar with Pn. Nevertheless, Ci had a contrary tendency. Under NaCl stress, Ci was much lower than the control group (*P* < 0.05), while Ci of PP groups with different MWs decreased as well compared to the salt stress group. This result indicated that PP relieved the stomata closure due to salt stress and promoted the utilization of CO_2_. The alleviating of stomatal closure may also benefit from the regulation of ions in stomata guard cells. Moreover, DPP1 promoted the photosynthesis is much efficiently more than other groups when in comparison with NaCl-stressed plants.

**Table 3:** Effects of four degraded (DPP1,2,3,4) and natural polysaccharides (PP) from *P. yezoensis* on growth parameters of wheat seedlings.

	Pn (μmol m^-2^s^-1^)	E (mol m^-2^s^-1^)	Gs (mol m^-2^s^-1^)	Ci (μmol m^-2^s^-1^)
Control	14.96 ± 1.44^d^	11.21 ± 1.2^a^	1.19 ± 0.13^a^	361.90 ± 4.53^a^
NaCl stress	10.36 ± 1.64^e^	8.04 ± 0.64^d^	0.60 ± 0.09^c^	333.30 ± 6.77^b^
DPP1 + NaCl stress	23.68 ± 3.30^a^	10.46 ± 1.70^ab^	0.86 ± 0.15^b^	316.80 ± 13.27^c^
DPP2 + NaCl stress	20.09 ± 3.47^b^	9.70 ± 1.68^bc^	0.83 ± 0.15^b^	316.10 ± 20.55^c^
DPP3 + NaCl stress	19.96 ± 2.23^b^	9.71 ± 1.6^bc^	0.79 ± 0.14^b^	317.90 ± 10.73^c^
DPP4 + NaCl stress	17.45 ± 2.24^c^	8.97 ± 1.97^cd^	0.79 ± 0.09^b^	318.80 ± 18.72^c^
PP + NaCl stress	16.05 ± 2.48^cd^	8.89 ± 1.36^cd^	0.75 ± 0.11^b^	318.40 ± 14.47^c^

*Values are mean ± SD of 10 replicates. Different letters indicate significant differences at P < 0.05.*

#### Soluble Sugar Content

**FIGURE 4:**
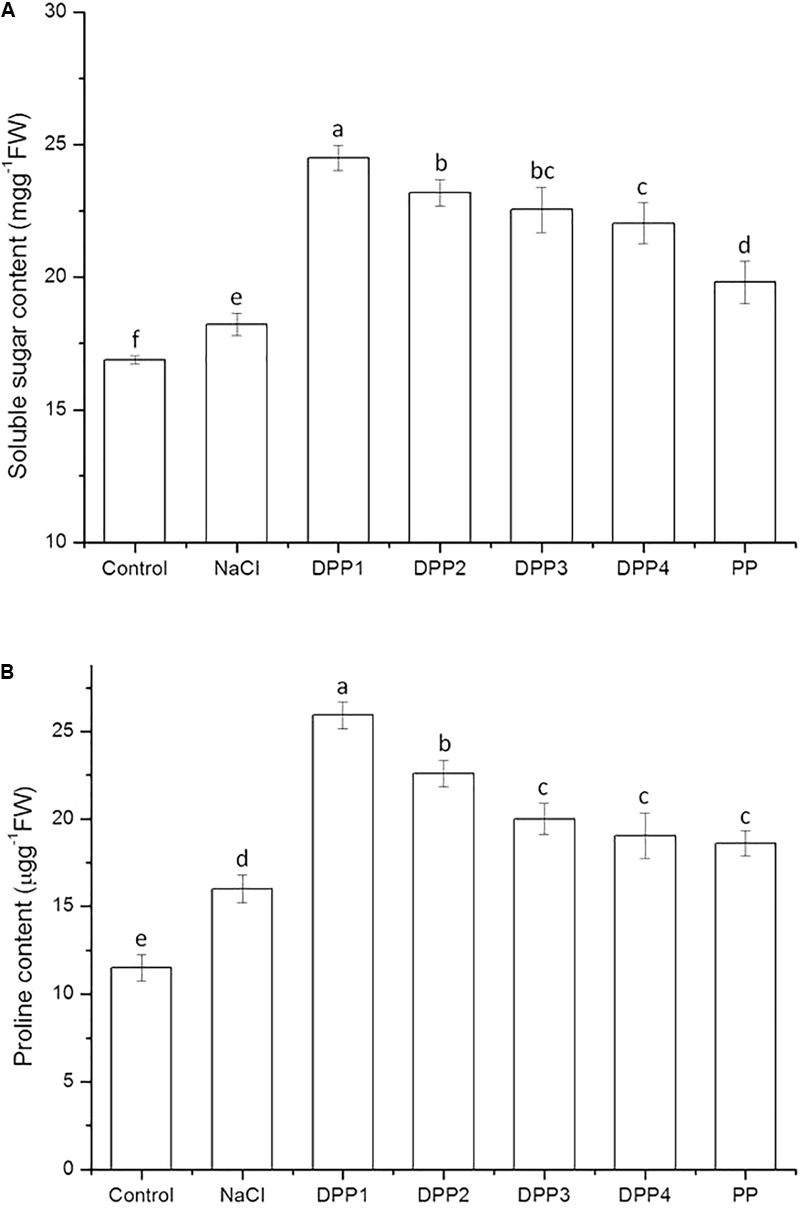
Effect of four degraded (DPP1,2,3,4) and natural polysaccharides (PP) from *P. yezoensis* on soluble sugar **(A)** and proline content **(B)** in wheat seedlings. Values are the mean ± SD of three replicates. Different letters indicate significant differences at *P* < 0.05.

The effect of PPs of different MW on the soluble sugar content of wheat seedlings is shown in **Figure 4A**. The results indicated that the soluble sugar content significantly increased in plants under salt stress relative to the control (*P* < 0.05). In wheat seedlings treated with exogenous PPs, the soluble sugar content increased by 34.4, 27.2, 23.7, 20.9, and 8.7%, respectively (*P* < 0.05) compared with the salt-stressed plants. The results showed that DPP1 more strongly induced soluble sugar production than the other DPPs and PP (*P* < 0.05).

Under salt stress, proteins and other macromolecules rapidly decompose. In the process, plant cell membranes are damaged and imbalances in osmotic pressure occur. Sugars act as osmoregulators and reduce membrane permeability. They lower the water potential in cells and stabilize membranes (Cao et al., 2014). The results showed that the content of soluble sugar increased in plants under NaCl stress (**Figure 4A**). The fact that the soluble sugar content increased in wheat seedlings treated with different PPs suggested that soluble sugar can help maintain osmotic balance and stabilize cell membranes in plants.

#### Proline Content

As shown in **Figure 4B**, the proline content in wheat seedling leaves increased by 39.3% (*P* < 0.05) relative to the control in response to the 10-days NaCl treatment. There was a significant increase in the proline content of wheat seedlings treated with PPs. After 10 days salt stress, PPs increased the proline content by 61.9, 41.1, 24.9, 19.0, and 16.2%, respectively (*P* < 0.05) compared with the NaCl stress group. The results showed that DPP1 more strongly promoted proline production and accumulation in the plants than the other groups (*P* < 0.05).

Under abiotic stress, low MW organic compounds, such as proline, betaine, and free amino acids were synthesized in the cytoplasm, to prevent cytoplasm dehydration caused by salt stress (Zeng et al., 2013). Proline not only occupies a compatible osmolyte and osmoprotectant under abiotic stresses, it also regulates osmotic potential, stabilizes cellular structure, and reduces damage to the photosynthetic apparatus. Zhang Z. et al. (2017) indicated that proline metabolic pathways exhibit significant differences during the salt stress response. Proline induces the expression of salt stress-responsive proteins, consequently increases the adaptation of plant to salt stress. The protective effect of proline may be explained by its ability to induce antioxidant enzymes to scavenge reactive oxygen species (ROS). There was a report that exogenous proline alleviated the oxidative damage by reducing H_2_O_2_ and lipid peroxidation levels and by increasing antioxidant activity (Ben Ahmed et al., 2010). The results of the present study showed that salt stress induced proline biosynthesis in salt-stressed plants to resist the osmotic imbalance caused by salt stress. This may be a stress reaction for plant to salt stress. PPs further increased the proline content in salt-stressed wheat seedlings in order to counteract the salt-induced osmotic stress. Most of all, the proline content in DPP1 group was obviously higher than that of other groups, indicated that the wheat seedlings of DPP1 group had stronger osmotic regulation ability.

#### Antioxidant Enzymes Activities

**FIGURE 5:**
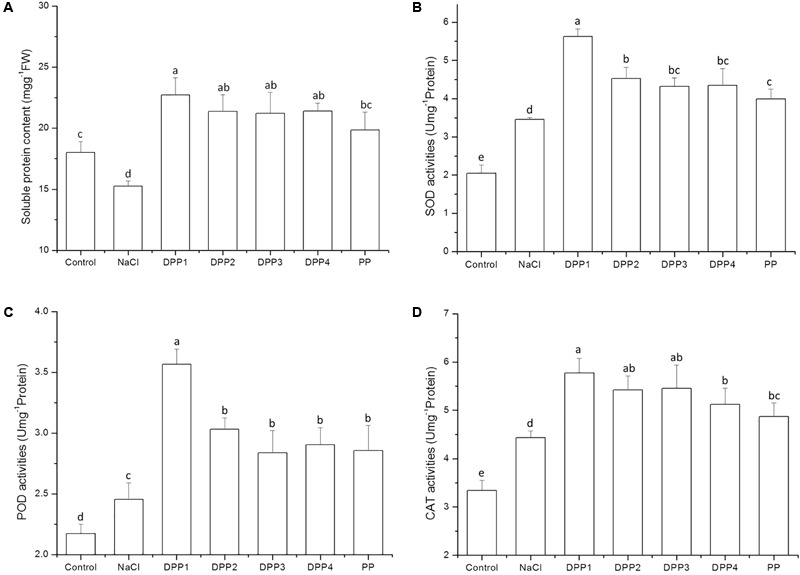
Effect of four degraded (DPP1,2,3,4) and natural polysaccharides (PP) from *P. yezoensis* on soluble protein contents **(A)**, SOD **(B)**, POD **(C)**, and CAT **(D)** activities in wheat seedlings. Values are the mean ± SD of three replicates. Different letters indicate significant differences at *P* < 0.05.

The results showed that soluble protein content decreased after 10 days salt stress. By that time, it was significantly lower than that of the control (*P* < 0.05). In wheat seedlings treated with exogenous PPs, the soluble protein content increased by 49.0, 40.2, 39.2, 40.4, and 30.1%, respectively (*P* < 0.05) relative to the negative control (**Figure 5**). There was, however, no significant difference in soluble protein content among the four DPP groups. Similarly, the application of PPs increased the POD activities by 45.3, 23.5, 15.6, 18.4, and 16.4% (*P* < 0.05) relative to the salt-stress treatment. Moreover, the SOD and POD activities in the DPP1 treatment were significantly higher than those in the other groups (*P* < 0.05).

In the present study, salt stress increased the SOD, POD, and CAT activities (**Figures 5B–D**) compared to the control. Compared with the control, the SOD activity rose to 69.1% after 10 days salt stress treatment (*P* < 0.05). Treatment with the various PPs further increased SOD activity. After 10 days NaCl stress, PPs increased the SOD activities by 62.9, 31.1, 25.2, 25.8, and 15.6%, respectively (*P* < 0.05) compared with the NaCl stress group. Similarly, PPs increased the POD activities by 45.3, 23.5, 15.6, 18.4, and 16.4%, respectively (*P* < 0.05) relative to the negative control. Moreover, the activities of SOD and POD in the DPP1 treatment were significantly higher than those in the other groups (*P* < 0.05). A similar trend was observed and recorded for the CAT activity but the levels of this enzyme did not significantly differ among the DPP1, DPP2, and DPP3 treatments.

Salinity-induced crop yield loss is a consequence of imbalances in mineral nutrients concentrations and osmotic effects which trigger the overproduction of ROS (Mittler et al., 2004). Under normal physiological conditions, ROS are constantly generated from aerobic metabolism in chloroplasts, mitochondria, and peroxisomes. Their overproduction, however, causes oxidative damage to lipids, proteins, and nucleic acids, and alters membrane permeability (Mittler et al., 2004). Adaptations which regulate ROS generation in plants may effectively defend them against oxidative damage and increase their stress tolerance.

The accumulation and activity of antioxidant enzymes help inhibit membrane protein and lipid peroxidation. There have been many studies to prove that SOD, POD, and CAT are strongly associated with salt resistance in plants (Ma et al., 2012; Rasool et al., 2012; Qiu et al., 2014). SOD detoxifies $O_{2}^{•}$ by forming H_2_O_2_. The latter is also toxic and must be eliminated by the concerted actions of CAT and POD. In the present study, salt stress induced SOD, POD, and CAT activities to levels significantly greater than those of the control. Plants receiving PPs under NaCl stress also showed relative increases in their SOD, POD, and CAT activities. The results indicated that PPs effectively induce ROS scavenging in wheat seedlings by modulating their antioxidant enzymes activities. Therefore, PPs may enhance defense responses in plants under salt stress. In general, DPP1 (3.2 kDa) was significantly more effective at inducing antioxidant activity and ROS scavenging than the other PP and DPPs.

#### Na^+^ and K^+^ Accumulation in Different Tissues of Wheat Seedlings

**FIGURE 6:**
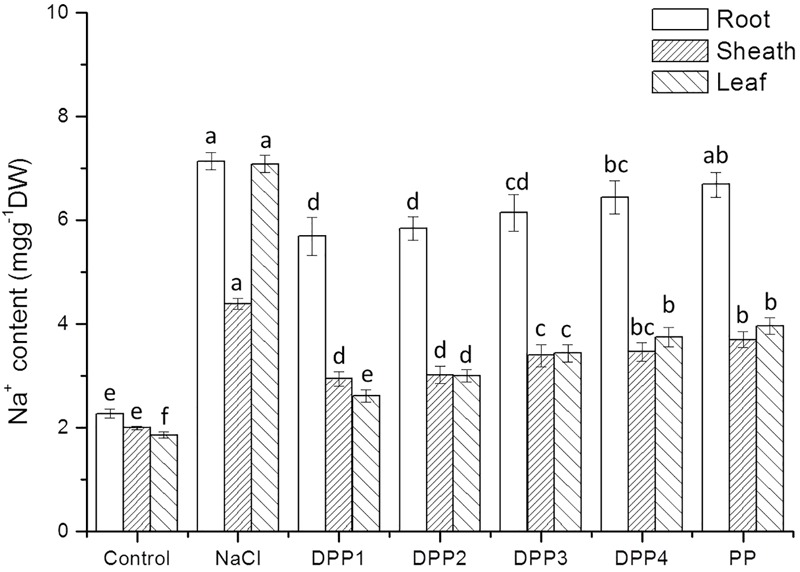
Effect of four degraded (DPP1,2,3,4) and natural polysaccharides (PP) from *P. yezoensis* on Na^+^ contents of root, sheath, and leaf in wheat seedlings. Values are the mean ± SD of three replicates. Different letters indicate significant differences at *P* < 0.05.

**FIGURE 7:**
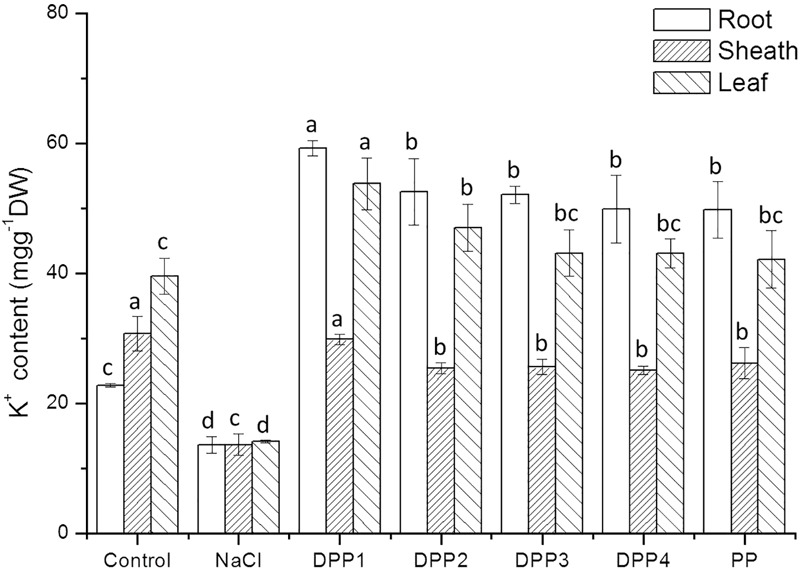
Effect of four degraded (DPP1,2,3,4) and natural polysaccharides (PP) from *P. yezoensis* on K^+^ contents of root, sheath, and leaf in wheat seedlings. Values are the mean ± SD of three replicates. Different letters indicate significant differences at *P* < 0.05.

**FIGURE 8:**
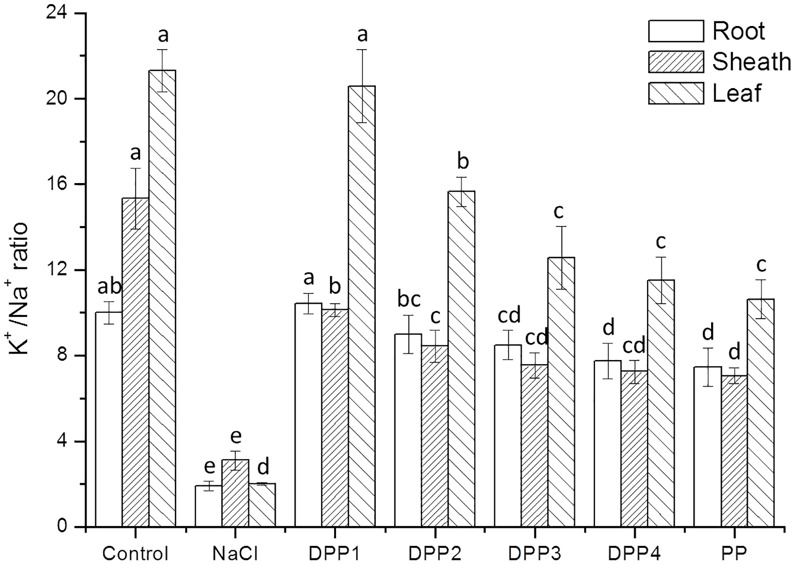
Effect of four degraded (DPP1,2,3,4) and natural polysaccharides (PP) from *P. yezoensis* on K^+^/Na^+^ ratio of root, sheath, and leaf in wheat seedlings. Values are the mean ± SD of three replicates. Different letters indicate significant differences at *P* < 0.05.

The results showed that Na^+^ content increased highly in different tissues of wheat seedlings after 10 days salt stress. In the root, sheath, and leaf, the increase in Na^+^ content of salt stressed plants was 3.1, 2.2, 3.8-times, respectively, higher than that of the control (*P* < 0.05, **Figure 6**). But there is a reverse trend in K^+^ content (**Figure 7**). The K^+^ content in the root, sheath, and leaf of salt stressed plants decreased by 40.5, 55.7, and 64.3%, respectively (*P* < 0.05). The Na^+^ contents in plant tissues of different polysaccharide treatment groups were lower than that of salt stress group, but still higher than that of control group. The results showed that Na^+^ accumulated in different tissues of plants under salt stress, especially in roots. In wheat seedlings treated with exogenous PPs, the K^+^ content increased 3.4-, 2.9-, 2.8-, 2.7-, and 2.7-fold relative to the negative control (**Figure 7**). Similarly, the application of PPs increased the K^+^ content in sheath and leaf compared to the salt-stress treatment. Consequently, a higher K^+^/Na^+^ ratio was observed in the root, sheath, and leaf of PP treated plants (**Figure 8**).

The ability to restrict the transport and accumulation of Na^+^ in the leaves is the most important adaptation of plant to salt stress (Mekawy et al., 2015). In this study, a large amount of Na^+^ was accumulated in roots of all the salt stressed plants. However, treated with exogenous PPs, leaf Na^+^ content was significantly lower compared with the salt stressed group. Thus, wheat seedlings treated with DPPs excluded Na^+^ selectively from leaves, the source tissue for photosynthesis (Lekshmy et al., 2015). Under salt stress, plants suffer from K^+^ deficiencies stemming from the competitive inhibition of its uptake by Na^+^, and this often leads to high Na^+^/K^+^ ratios that disrupt cellular homeostasis (Mekawy et al., 2015). In this work, the K^+^ immense accumulation in PPs treated plants was accompanied by a higher K^+^/Na^+^ ratio, which contributed to the salt tolerance. Moreover, the K^+^/Na^+^ ratio in DPP1 treated plants was much higher than that of other groups.

#### Expression of Genes Encoding Na^+^/K^+^ Transporter

**FIGURE 9:**
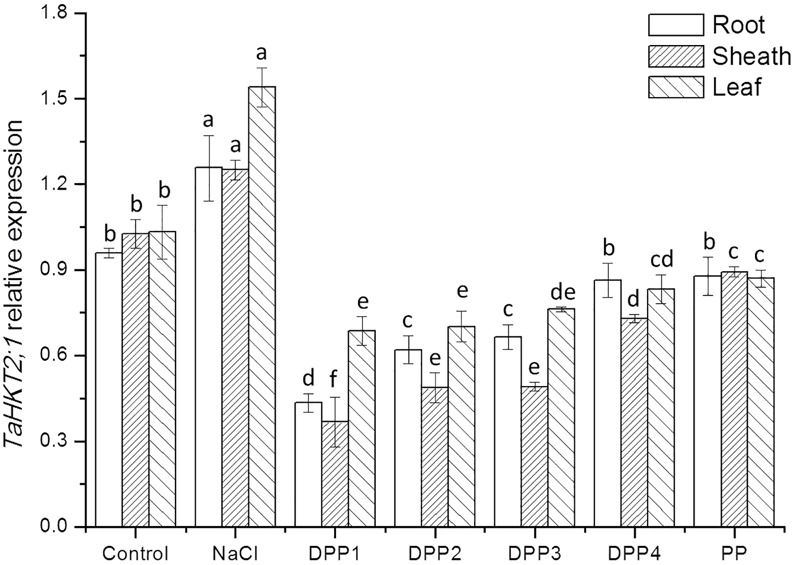
Effect of four degraded (DPP1,2,3,4) and natural polysaccharides (PP) from *P. yezoensis* on *TaHKT2;1* expression of root, sheath, and leaf in wheat seedlings. Values are the mean ± SD of three replicates. Different letters indicate significant differences at *P* < 0.05.

**FIGURE 10:**
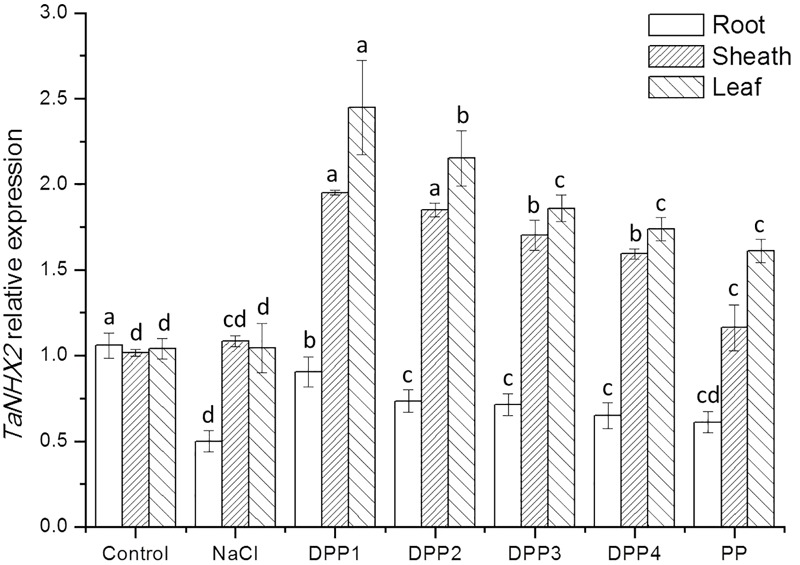
Effect of four degraded (DPP1,2,3,4) and natural polysaccharides (PP) from *P. yezoensis* on *TaNHX2* expression of root, sheath, and leaf in wheat seedlings. Values are the mean ± SD of three replicates. Different letters indicate significant differences at *P* < 0.05.

**FIGURE 11:**
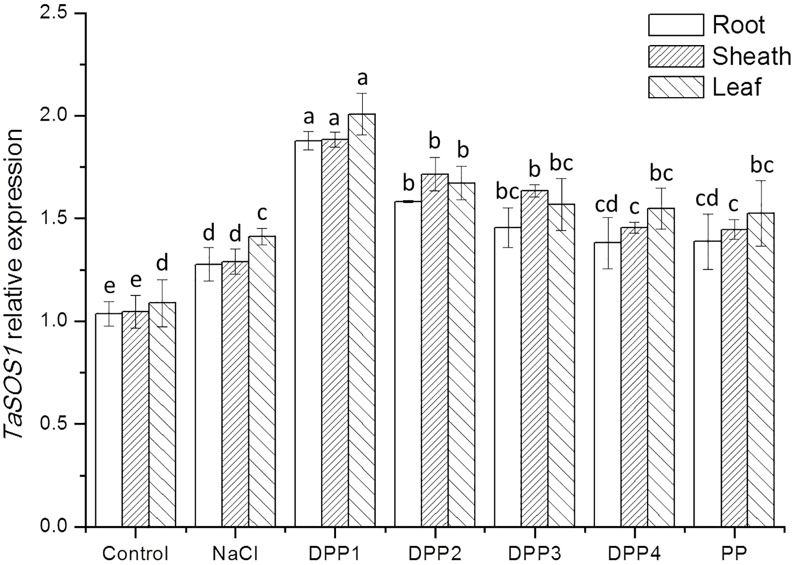
Effect of four degraded (DPP1,2,3,4) and natural polysaccharides (PP) from *P. yezoensis* on *TaSOS1* expression of root, sheath, and leaf in wheat seedlings. Values are the mean ± SD of three replicates. Different letters indicate significant differences at *P* < 0.05.

To determine the mechanisms underlying differential Na^+^ and K^+^ accumulation in salt stressed and PPs treated plants, genes expression of Na^+^ and K^+^ transporter were analyzed. The analysis showed that compared with the control, salt stress induced higher transcript levels of the *TaHKT2;1* gene (**Figure 9**). While treated with PPs, the expression of *TaHKT2;1* was down regulated in the root, sheath, and leaf significantly (**Figure 9**). By contrast, the analysis of *TaNHX2* revealed that under salt stress, the *TaNHX2* expression was obviously down regulated in the root of the salt stressed plants, but it there was no significant difference in sheath and leaf (**Figure 10**). After the application of different PPs, the *TaNHX2* expression was still down regulated in the root, while the gene was up regulated in the sheath and leaf. In the leaf, it was observed to be 2.3-, 2.1-, 1.8-, 1.7-, and 1.5-fold in the PPs treated plants, respectively, compared to the salt stress plants. There was a similar trend for the *TaNHX2* expression in sheath. The quantitative analysis of the *TaSOS1* showed that compared with the control group, salt stress induced higher transcript levels of the *TaSOS1* gene (**Figure 11**). While treated with PPs, the expression of *TaSOS1* was up regulated significantly in the root, sheath, and leaf (**Figure 11**). Moreover, the transcript levels of *TaSOS1* treated with DPP1 were significantly higher than those of the other groups (*P* < 0.05).

High-affinity potassium transporters (HKTs) have been reported to function as Na^+^/K^+^ symporters as well as selective Na^+^ uniporters (Horie et al., 2009). In the bread wheat, *TaHKT2;1* has been identified and presumed to function in Na^+^ uptake from the soil (Ariyarathna et al., 2014). The expression analysis of *TaHKT2;1* in the contrasting wheat genotypes has been studied previously (Kumar et al., 2017). The expression of *TaHKT2;1* was observed to upregulate in the shoots of the salt-sensitive genotype, while it was downregulated in the salt-tolerant genotype. It shows that the inhibition of gene expression was related to its salt resistance. In this study, salt stress induced higher transcript levels of the *TaHKT2;1* gene (**Figure 9**). While treated with PPs, the expression of *TaHKT2;1* was down regulated in the root significantly. The results indicated that the role of *TaHKT2;1* in restricting uptake Na^+^ from the soil to the roots and consequently a lower Na^+^ level and Na^+^/K^+^ ratio in the leaves. Recently, Kumar et al. (2017) claimed that under salt stress the increase in cytosine methylation down regulated the expression of *TaHKT 2;1* and *TaHKT2;3* in the salt-tolerant wheat, thereby improved its salt-tolerance ability.

In addition to limiting the entry of Na^+^ into cells, the mechanisms for salt tolerance also includes transporting Na^+^ out of cells and compartmentalizing Na^+^ into vacuoles (Yamaguchi et al., 2013). The Salt Overly Sensitive 1 (SOS1), a plasma membrane Na^+^/K^+^ antiporter mediates cytosolic Na^+^ efflux at the root and regulates Na^+^ transportation from root to shoot thereby maintaining appropriated K^+^/Na^+^ ratio in leaves, where the photosynthesis take place (Qiu et al., 2002). The Na^+^ compartmentation into the vacuoles depends on the activity of endosomal Na^+^/H^+^ antiporters such as NHX (Gaxiola et al., 2001). It was reported that expression levels of *SOS1* and *NHX1* were significantly higher in salt tolerant wheat genotypes under 200 mM NaCl stress, and consequently correlated with improved sodium exclusion and lower Na^+^/K^+^ ratio (Lekshmy et al., 2015). In this work, after application of PPs, the overexpression of *TaSOS1* and *TaNHX2* was markly upregulated in the wheat seedlings which were much more resistant to high concentrations of NaCl. In general, the transcript levels of *TaSOS1* and *TaNHX2* genes treated with DPP1 were significantly higher than those of the other groups (*P* < 0.05). Our results indicated that DPP1 can effectively coordinate the efflux and regionalization of Na^+^, alleviate the salt stress damage.

### Effects of Polysaccharides From *Pyropia yezoensis* With Different MWs on the Plants’ Defense Response Under Salt Stress

Polysaccharides from *P. yezoensis* are high-MW polysaccharides with high viscosity. This physical property may limit its practical application in agriculture, pharmaceutical, and other industries. Many studies demonstrated that polysaccharide MW distributions significantly influence their biological functions. Polysaccharides with relatively lower MWs have been reported to have higher antioxidant effects than those with higher MWs (Qi et al., 2006; Zha et al., 2016). Li et al. (2013) prepared six representative sulphated polysaccharides (446.5, 247.0, 76.1, 19.0, 5.0, and 3.1 kDa) using the microwave-assisted acid hydrolysis method and investigated their antioxidant activities. Samples with high MW inhibited superoxide radicals more effectively than did lower-MW polysaccharides. In contrast, samples with low MW more effectively inhibited hydroxyl radicals than those with higher MW. Nevertheless, Hou et al. (2012) reported that the relationship between the MW of fucoidans (1.0, 3.8, 8.3, 13.2, 35.5, 64.3, and 144.5 kDa) and their antioxidant activities is non-linear. The 3.8, 1.0, and >8.3 kDa samples had relatively superior hydroxyl radical scavenging activity, reducing power, and superoxide anion scavenging activity, respectively.

Sulfate content significantly influences the superoxide anion scavenging ability of fucoidans (Rocha de Souza et al., 2007). In the present study, all the samples had similar sulfate levels, so this factor could be ignored. The results suggest that lower-MW samples (DPP1, 3.2 kDa) are the most effective at protecting wheat seedlings against salt stress. The close correlation between the activity and the MW of polysaccharides reveals that the former is dependent on a specific chemical structure. Polysaccharides with different MW may form different configurations that interact with elicitor receptors on plant cell membranes. Polysaccharides that are too small or large are often inactive. The appropriate degree of polymerization will effectively induce a regulatory response. The capacity of functional oligosaccharides produced by alginase was studied on the growth promoting of *Brassica campestris* L. under salt stress. The plant growth experiment suggested that low MW oligosaccharides were more effective in stimulating root elongation and relieving salt stress (Tang et al., 2011). Seven chitooligomers (COSs) with determined degrees of polymerization (chitotetraose to chitooctaose, DP 8–10, DP 10–12) were applied to explore the relationship between the degrees of polymerization of COSs and its effect on the tolerance of wheat seedlings to salt stress. The results suggested that chitohexaose, chitoheptaose, and chitooctaose exhibited stronger activity compared with other COS samples, which suggested that its activity had a closely relationship with its degrees of polymerization (Zhang X. et al., 2017). Ulteriorly, Wei et al. (2009) investigated the effects of chitooligomers (COS) COS5 and COS6 *in vivo* and *in vitro* on the expression of the gene coding for the CR3 cell surface receptor. The results showed that both COS5 and COS6 promote the expression of CR3 mRNA, and this effect was stronger in response to COS6 than COS5. Both of these activated phagocytes and enhanced antibody transmission by binding to the CR3 receptor. COS6 has a higher MW and more exposed amino glucose groups than COS5. Consequently, relative to COS5, COS6 has more active binding sites available to combine with the CR3 receptor surfaces of macrophages and lymphocytes. What is more, COS6 can change the configuration of the CR3 receptor and increase its substrate affinity. The function of these plasma membrane proteins is related to the perception of the elicitor signal. Nevertheless, the mechanisms underlying elicitor recognition, including polysaccharides and signal transduction, require further investigation.

## Conclusion

This study developed an efficient method for the degradation of *P. yezoensis* polysaccharide using microwave-assisted acid hydrolysis. Microwave exposures significantly accelerated the reaction rate. Polysaccharides with different MW could be obtained by strictly controlling the reaction conditions. Representative polysaccharides with different MW (3.2, 10.5, 29.0, and 48.8 kDa) were prepared and the relationship between their MW and their effects on plant salt tolerance was investigated. The results showed that degradation of *P. yezoensis* polysaccharide could protect plants from salt stress damage by regulating antioxidant enzyme activities and the content of permeable substances. The expression level examination analysis of several Na^+^/K^+^ transporter genes indicated that DPPs could restrict the transport and accumulation of Na^+^ in the wheat seedlings, maintain a higher K^+^/Na^+^ ratio, thereby protect plants from the damage of salt stress. Furthermore, the effect of polysaccharides on the tolerance of wheat seedlings to salt stress was closely correlated with polysaccharide MW.

## Author Contributions

PZ: Conceived the study and wrote the manuscript. LM and HZ: Preparation of polysaccharide. CJ and YY: Chemical analysis of polysaccharide. XL and YL: Study the effect of polysaccharides on wheat seedlings under salt stress. CZ and YL: Reviewed and edited the manuscript.

## Conflict of Interest Statement

The authors declare that the research was conducted in the absence of any commercial or financial relationships that could be construed as a potential conflict of interest.

## Supplementary Material

FIGURE S1 The Mw of four degraded (DPP1,2,3,4) and natural polysaccharides (PP) from *P. yezoensis*.

TABLE S1 Reaction conditions of degraded polysaccharides from *P. yezoensis*.

TABLE S2 Primers used for quantitative real-time RT-PCR.

## References

[B1] Ariyarathna H. A. Ul-Haq T. Colmer T. D. Francki M. G. (2014). Characterization of the multigene family TaHKT 2;1 in bread wheat and the role of gene members in plant Na(+) and K(+) status. *BMC Plant Biol.* 14:159. 10.1186/1471-2229-14-159 24920193 PMC4079177

[B2] Ben Ahmed C. Ben Rouina B. Sensoy S. Boukhriss M. Ben Abdullah F. (2010). Exogenous Proline Effects on Photosynthetic Performance and Antioxidant Defense System of Young Olive Tree. *J. Agric. Food Chem.* 58 4216–4222. 10.1021/jf9041479 20210359

[B3] Bose J. Rodrigo-Moreno A. Shabala S. (2014). ROS homeostasis in halophytes in the context of salinity stress tolerance. *J. Exp. Bot.* 65 1241–1257. 10.1093/jxb/ert430 24368505

[B4] Cao Y.-Y. Yang M.-T. Li X. Zhou Z.-Q. Wang X.-J. Bai J.-G. (2014). Exogenous sucrose increases chilling tolerance in cucumber seedlings by modulating antioxidant enzyme activity and regulating proline and soluble sugar contents. *Sci. Hortic.* 179 67–77. 10.1016/j.scienta.2014.09.016

[B5] Gaxiola R. A. Li J. Undurraga S. Dang L. M. Allen G. J. Alper S. L. (2001). Drought- and salt-tolerant plants result from overexpression of the AVP1 H+-pump. *Proc. Natl. Acad. Sci. U.S.A.* 98 11444–11449. 10.1073/pnas.191389398 11572991 PMC58749

[B6] Horie T. Hauser F. Schroeder J. I. (2009). HKT transporter-mediated salinity resistance mechanisms in *Arabidopsis* and monocot crop plants. *Trends Plant Sci.* 14 660–668. 10.1016/j.tplants.2009.08.009 19783197 PMC2787891

[B7] Hou Y. Wang J. Jin W. Zhang H. Zhang Q. (2012). Degradation of *Laminaria japonica* fucoidan by hydrogen peroxide and antioxidant activities of the degradation products of different molecular weights. *Carbohydr. Polym.* 87 153–159. 10.1016/j.carbpol.2011.07.031 34662944

[B8] Huang C. Wang D. Sun L. Wei L. (2015). Effects of exogenous salicylic acid on the physiological characteristics of *Dendrobium officinale* under chilling stress. *Plant Growth Regul.* 79 199–208. 10.1007/s10725-015-0125-z

[B9] Kumar S. Beena A. S. Awana M. Singh A. (2017). Salt-induced tissue-specific cytosine methylation downregulates expression of HKT genes in contrasting wheat (*Triticum aestivum* L.) genotypes. *DNA Cell Biol.* 36 283–294. 10.1089/dna.2016.3505 28384069 PMC5385449

[B10] Lekshmy S. Sairam R. K. Chinnusamy V. Jha S. K. (2015). Differential transcript abundance of salt overly sensitive (SOS) pathway genes is a determinant of salinity stress tolerance of wheat. *Acta Physiol. Plant.* 37:169 10.1007/s11738-015-1910-z

[B11] Li B. Liu S. Xing R. Li K. Li R. Qin Y. (2013). Degradation of sulfated polysaccharides from *Enteromorpha prolifera* and their antioxidant activities. *Carbohydr. Polym.* 92 1991–1996. 10.1016/j.carbpol.2012.11.088 23399249

[B12] Li H. Chang J. Chen H. Wang Z. Gu X. Wei C. (2017). Exogenous melatonin confers salt stress tolerance to watermelon by improving photosynthesis and redox homeostasis. *Front. Plant Sci.* 8:295. 10.3389/fpls.2017.00295 28298921 PMC5331065

[B13] Ma L. Li Y. Yu C. Wang Y. Li X. Li N. (2012). Alleviation of exogenous oligochitosan on wheat seedlings growth under salt stress. *Protoplasma* 249 393–399. 10.1007/s00709-011-0290-5 21626287

[B14] Ma X. Zheng J. Zhang X. Hu Q. Qian R. (2017). Salicylic acid alleviates the adverse effects of salt stress on *Dianthus superbus* (Caryophyllaceae) by activating photosynthesis, protecting morphological structure, and enhancing the antioxidant system. *Front. Plant Sci.* 8:600. 10.3389/fpls.2017.00600 28484476 PMC5399920

[B15] Mehta P. Jajoo A. Mathur S. Bharti S. (2010). Chlorophyll a fluorescence study revealing effects of high salt stress on Photosystem II in wheat leaves. *Plant Physiol. Biochem.* 48 16–20. 10.1016/j.plaphy.2009.10.006 19932973

[B16] Mekawy A. M. Assaha D. V. Yahagi H. Tada Y. Ueda A. Saneoka H. (2015). Growth, physiological adaptation, and gene expression analysis of two Egyptian rice cultivars under salt stress. *Plant Physiol. Biochem.* 87 17–25. 10.1016/j.plaphy.2014.12.007 25532120

[B17] Mishra P. Bhoomika K. Dubey R. S. (2013). Differential responses of antioxidative defense system to prolonged salinity stress in salt-tolerant and salt-sensitive Indica rice (*Oryza sativa* L.) seedlings. *Protoplasma* 250 3–19. 10.1007/s00709-011-0365-3 22194018

[B18] Mittler R. Vanderauwera S. Gollery M. Van Breusegem F. (2004). Reactive oxygen gene network of plants. *Trends Plant Sci.* 9 490–498. 10.1016/j.tplants.2004.08.009 15465684

[B19] Mohibbullah M. Bhuiyan M. M. Hannan M. A. Getachew P. Hong Y. K. Choi J. S. (2015). The edible red alga *Porphyra yezoensis* promotes neuronal survival and cytoarchitecture in primary hippocampal neurons. *Cell. Mol. Neurobiol.* 36 669–682. 10.1007/s10571-015-0247-x 26259718 PMC11482408

[B20] Qi H. Zhao T. Zhang Q. Li Z. Zhao Z. Xing R. (2006). Antioxidant activity of different molecular weight sulfated polysaccharides from *Ulva pertusa* Kjellm (Chlorophyta). *J. Appl. Phycol.* 17 527–534. 10.1007/s10811-005-9003-9

[B21] Qian L. Zhou Y. Ma J.-X. (2014). Hypolipidemic effect of the polysaccharides from *Porphyra yezoensis*. *Int. J. Biol. Macromol.* 68 48–49. 10.1016/j.ijbiomac.2014.04.004 24736124

[B22] Qiu Q. S. Guo Y. Dietrich M. A. Schumaker K. S. Zhu J. K. (2002). Regulation of SOS1, a plasma membrane Na+/H+ exchanger in *Arabidopsis thaliana*, by SOS2 and SOS3. *Proc. Natl. Acad. Sci. U.S.A.* 99 8436–8441. 10.1073/pnas.122224699 12034882 PMC123085

[B23] Qiu Z. Guo J. Zhu A. Zhang L. Zhang M. (2014). Exogenous jasmonic acid can enhance tolerance of wheat seedlings to salt stress. *Ecotoxicol. Environ. Saf.* 104 202–208. 10.1016/j.ecoenv.2014.03.014 24726929

[B24] Rasool S. Ahmad A. Siddiqi T. O. Ahmad P. (2012). Changes in growth, lipid peroxidation and some key antioxidant enzymes in chickpea genotypes under salt stress. *Acta Physiol. Plant.* 35 1039–1050. 10.1007/s11738-012-1142-4

[B25] Rivero R. M. Mestre T. C. Mittler R. Rubio F. Garcia-Sanchez F. Martinez V. (2014). The combined effect of salinity and heat reveals a specific physiological, biochemical and molecular response in tomato plants. *Plant Cell Environ.* 37 1059–1073. 10.1111/pce.12199 24028172

[B26] Rocha de Souza M. C. Marques C. T. Guerra Dore C. M. Ferreira Da Silva F. R. Oliveira Rocha H. A. (2007). Antioxidant activities of sulfated polysaccharides from brown and red seaweeds. *J. Appl. Phycol.* 19 153–160. 10.1007/s10811-006-9121-z 19396353 PMC2668642

[B27] Sangha J. S. Ravichandran S. Prithiviraj K. Critchley A. T. Prithiviraj B. (2010). Sulfated macroalgal polysaccharides λ-carrageenan and ι-carrageenan differentially alter *Arabidopsis thaliana* resistance to *Sclerotinia sclerotiorum*. *Physiol. Mol. Plant Pathol.* 75 38–45. 10.1016/j.pmpp.2010.08.003

[B28] Stadnik M. J. de Freitas M. B. (2014). Algal polysaccharides as source of plant resistance inducers. *Trop. Plant Pathol.* 39 111–118. 10.1590/S1982-56762014000200001

[B29] Sudhir P. M. Murthy S. D. S. (2004). Effects of salt stress on basic processes of photosynthesis. *Photosynthetica* 42 481–486. 10.1007/S11099-005-0001-6

[B30] Tang J. Zhou Q. Chu H. Nagata S. (2011). Characterization of alginase and elicitor-active oligosaccharides from *Gracilibacillus* A7 in alleviating salt stress for *Brassica campestris* L. *J. Agric. Food Chem.* 59 7896–7901. 10.1021/jf201793s 21696216

[B31] Van Oosten M. J. Silletti S. Guida G. Cirillo V. Di Stasio E. Carillo P. (2017). A benzimidazole proton pump inhibitor increases growth and tolerance to salt stress in tomato. *Front. Plant Sci.* 8:1220. 10.3389/fpls.2017.01220 28769943 PMC5513968

[B32] Vera J. Castro J. Contreras R. A. González A. Moenne A. (2012). Oligo-carrageenans induce a long-term and broad-range protection against pathogens in tobacco plants (var. Xanthi). *Physiol. Mol. Plant Pathol.* 79 31–39. 10.1016/j.pmpp.2012.03.005

[B33] Wei X. Wang Y. Xiao J. Xia W. (2009). Separation of chitooligosaccharides and the potent effects on gene expression of cell surface receptor CR3. *Int. J. Biol. Macromol.* 45 432–436. 10.1016/j.ijbiomac.2009.07.003 19635497

[B34] Yamaguchi T. Hamamoto S. Uozumi N. (2013). Sodium transport system in plant cells. *Front. Plant Sci.* 4:410. 10.3389/fpls.2013.00410 24146669 PMC3797977

[B35] Yu X. Zhou C. Yang H. Huang X. Ma H. Qin X. (2015). Effect of ultrasonic treatment on the degradation and inhibition cancer cell lines of polysaccharides from *Porphyra yezoensis*. *Carbohydr. Polym.* 117 650–656. 10.1016/j.carbpol.2014.09.086 25498684

[B36] Zahra J. Nazim H. Cai S. Han Y. Wu D. Zhang B. (2014). The influence of salinity on cell ultrastructures and photosynthetic apparatus of barley genotypes differing in salt stress tolerance. *Acta Physiol. Plant.* 36 1261–1269. 10.1007/s11738-014-1506-z

[B37] Zeng J. Chen A. Li D. Yi B. Wu W. (2013). Effects of salt stress on the growth, physiological responses, and glycoside contents of *Stevia rebaudiana* Bertoni. *J. Agric. Food Chem.* 61 5720–5726. 10.1021/jf401237x 23711229

[B38] Zha S. Zhao Q. Zhao B. Ouyang J. Mo J. Chen J. (2016). Molecular weight controllable degradation of *Laminaria japonica* polysaccharides and its antioxidant properties. *J. Ocean Univ. China* 15 637–642. 10.1007/s11802-016-2943-7

[B39] Zhang M. Cao Y. Wang Z. Wang Z. Q. Shi J. Liang X. (2018). A retrotransposon in an HKT1 family sodium transporter causes variation of leaf Na(+) exclusion and salt tolerance in maize. *New Phytol.* 217 1161–1176. 10.1111/nph.14882 29139111

[B40] Zhang Q. Li N. Zhou G. Lu X. Xu Z. Li Z. (2003). In vivo antioxidant activity of polysaccharide fraction from *Porphyra haitanesis* (Rhodephyta) in aging mice. *Pharmacol. Res.* 48 151–155. 10.1016/s1043-6618(03)00103-8 12798667

[B41] Zhang S. Gan Y. Xu B. (2016). Application of plant-growth-promoting fungi trichoderma longibrachiatum t6 enhances tolerance of wheat to salt stress through improvement of antioxidative defense system and gene expression. *Front. Plant Sci.* 7:1405. 10.3389/fpls.2016.01405 27695475 PMC5023664

[B42] Zhang X. Li K. Liu S. Zou P. Xing R. Yu H. (2017). Relationship between the degree of polymerization of chitooligomers and their activity affecting the growth of wheat seedlings under salt stress. *J. Agric. Food Chem.* 65 501–509. 10.1021/acs.jafc.6b03665 28005356

[B43] Zhang Z. Mao C. Shi Z. Kou X. (2017). The amino acid metabolic and carbohydrate metabolic pathway play important roles during salt-stress response in tomato. *Front. Plant Sci.* 8:1231. 10.3389/fpls.2017.01231 28769946 PMC5511834

[B44] Zhao T. Zhang Q. Qi H. Zhang H. Niu X. Xu Z. (2006). Degradation of porphyran from *Porphyra haitanensis* and the antioxidant activities of the degraded porphyrans with different molecular weight. *Int. J. Biol. Macromol.* 38 45–50. 10.1016/j.ijbiomac.2005.12.018 16443266

[B45] Zhou C. Yu X. Zhang Y. He R. Ma H. (2012). Ultrasonic degradation, purification and analysis of structure and antioxidant activity of polysaccharide from *Porphyra yezoensis* Udea. *Carbohydr. Polym.* 87 2046–2051. 10.1016/j.carbpol.2011.10.026

[B46] Zou P. Li K. Liu S. He X. Zhang X. Xing R. (2016). Effect of sulfated chitooligosaccharides on wheat seedlings (*Triticum aestivum* L.) under salt stress. *J. Agric. Food Chem.* 64 2815–2821. 10.1021/acs.jafc.5b05624 26927620

[B47] Zou P. Li K. Liu S. Xing R. Qin Y. Yu H. (2015). Effect of chitooligosaccharides with different degrees of acetylation on wheat seedlings under salt stress. *Carbohydr. Polym.* 126 62–69. 10.1016/j.carbpol.2015.03.028 25933523

