# Supplementary figures and images for: Low-Molecular-Weightt Polysaccharides From Pyropia yezoensis Enhance Tolerance of Wheat Seedlings (Triticum aestivum L.) to Salt Stress
Source: Front Plant Sci. 2018 Apr 17;9:427. doi: 10.3389/fpls.2018.00427 (PMC5913351; doi:10.3389/fpls.2018.00427)

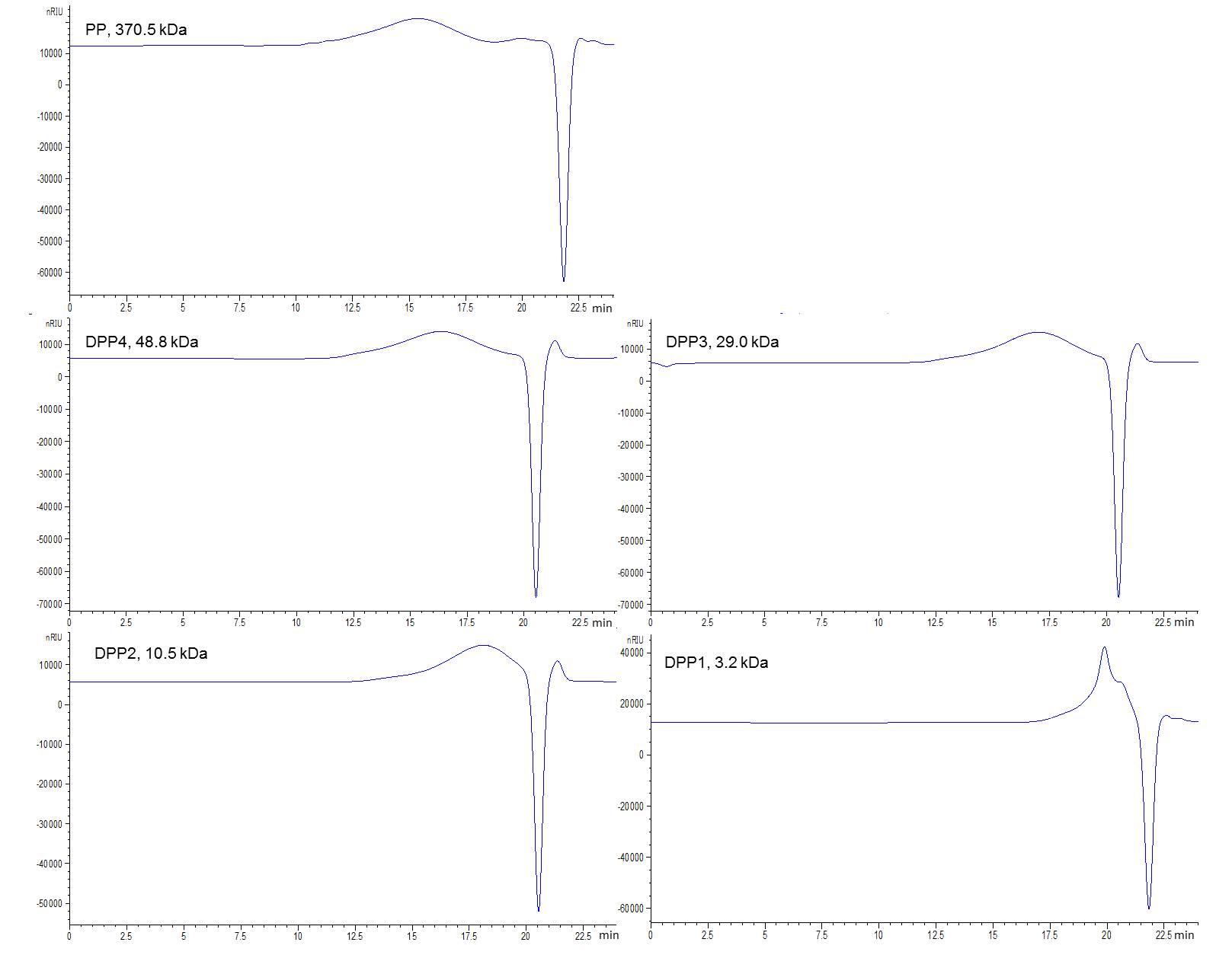

Supplement: FIGURE S1 — The Mw of four degraded (DPP1,2,3,4) and natural polysaccharides (PP) from P. yezoensis. [file Image_1.JPEG]
