# Supplementary material for: Low-Molecular-Weightt Polysaccharides From Pyropia yezoensis Enhance Tolerance of Wheat Seedlings (Triticum aestivum L.) to Salt Stress
Source: Front Plant Sci. 2018 Apr 17;9:427. doi: 10.3389/fpls.2018.00427 (PMC5913351; doi:10.3389/fpls.2018.00427)
Supplement: TABLE S1 — Reaction conditions of degraded polysaccharides from P. yezoensis. [file Table_1.DOCX]

Table S1. Reaction conditions of degraded polysaccharides from *P. yezoensis.*

| Sample | MW  (kDa) | reaction conditions | | | |
| --- | --- | --- | --- | --- | --- |
|  |  | HCl(M) | H_2_O_2_(%) | Tempreature(℃) | Time(min) |
| PP | 370.5 | - | - | - | - |
| DPP1 | 3.2 | 1.0 | 0.3 | 70 | 60 |
| DPP2 | 10.5 | 0.1 | 0.3 | 70 | 20 |
| DPP3 | 29.0 | 0.1 | 0.3 | 70 | 10 |
| DPP4 | 48.8 | 0.1 | 0.3 | 70 | 5 |

Table S2. Primers used for quantitative real-time RT-PCR.

| Gene | Accession | Primer pairs |
| --- | --- | --- |
| *TaNHX2* | AY040246 | F：TTCCAACCAGAACCAACCC  R：GTCCTTCATCGCTGAGACTTTT |
| *TaSOS1* | AY326952 | F：CGGAGGGTGGATTGAACGA  R：GCAGGGCGGTAGGAGAAGAT |
| *TaHKT2;1* | KR422358.1 | F：TATGTGATGAGTCGCAGCTTGAA  R：GCAACAAGAGGCCTGAATTCTTT |
| β-actin | AB181991 | F：CTCTGACAATTTCCCGCTCA  R：ACACGCTTCCTCATGCTATCC |
